# Bis(azido-κ*N*)(1,10-phenanthroline-κ^2^ *N*,*N*′)palladium(II)

**DOI:** 10.1107/S1600536812001201

**Published:** 2012-01-14

**Authors:** Kwang Ha

**Affiliations:** a School of Applied Chemical Engineering, The Research Institute of Catalysis, Chonnam National University, Gwangju 500-757, Republic of Korea

## Abstract

In the title complex, [Pd(N_3_)_2_(C_12_H_8_N_2_)], the Pd^II^ ion is four-coordinated in a slightly distorted square-planar environment by two N atoms of the chelating 1,10-phenanthroline (phen) ligand and two N atoms from two azide anions. The azido ligands are slightly bent with bond angles of 174.8 (4) and 174.5 (5)°. The complex mol­ecules are stacked in columns along the *a* axis and are connected by inter­molecular C—H⋯N hydrogen bonds, forming a three-dimensional network. In the columns, numerous inter­molecular π–π inter­actions between the six-membered rings are present, the shortest ring centroid–centroid distance being 3.607 (2) Å.

## Related literature

For the syntheses of [Pd*X*_2_(phen)] (*X* = Cl, Br, I or SCN), see: Cheng *et al.* (1977 ▶). For the crystal structures of [Pd*X*_2_(phen)] (*X* = Cl, Br or I), see: Ha (2010**a* ▶,*b* ▶,c* ▶).
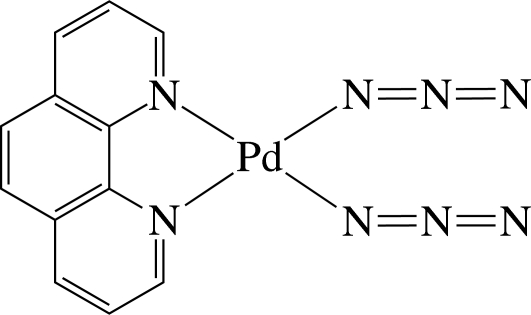


## Experimental

### 

#### Crystal data

[Pd(N_3_)_2_(C_12_H_8_N_2_)]
*M* *_r_* = 370.66
Orthorhombic, 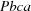

*a* = 7.0724 (3) Å
*b* = 18.3069 (7) Å
*c* = 19.1309 (7) Å
*V* = 2476.95 (17) Å^3^
*Z* = 8
Mo *K*α radiation
μ = 1.51 mm^−1^
*T* = 200 K
0.25 × 0.13 × 0.12 mm

#### Data collection

Bruker SMART 1000 CCD diffractometer
Absorption correction: multi-scan (*SADABS*; Bruker, 2000 ▶) *T*_min_ = 0.886, *T*_max_ = 1.000
17018 measured reflections
3058 independent reflections
2244 reflections with *I* > 2σ(*I*)
*R*_int_ = 0.040

#### Refinement

*R*[*F* ^2^ > 2σ(*F* ^2^)] = 0.033
*wR*(*F* ^2^) = 0.084
*S* = 1.09
3058 reflections
190 parameters
H-atom parameters constrained
Δρ_max_ = 1.20 e Å^−3^
Δρ_min_ = −0.62 e Å^−3^

### 

Data collection: *SMART* (Bruker, 2000 ▶); cell refinement: *SAINT* (Bruker, 2000 ▶); data reduction: *SAINT*; program(s) used to solve structure: *SHELXS97* (Sheldrick, 2008 ▶); program(s) used to refine structure: *SHELXL97* (Sheldrick, 2008 ▶); molecular graphics: *ORTEP-3* (Farrugia, 1997 ▶) and *PLATON* (Spek, 2009 ▶); software used to prepare material for publication: *SHELXL97*.

## Supplementary Material

Crystal structure: contains datablock(s) global. DOI: 10.1107/S1600536812001201/wm2583sup1.cif

Additional supplementary materials:  crystallographic information; 3D view; checkCIF report

## Figures and Tables

**Table:** 

Pd1—N1	2.038 (3)
Pd1—N2	2.040 (3)
Pd1—N3	2.012 (3)
Pd1—N6	2.013 (3)

**Table:** 

N1—Pd1—N2	81.20 (12)
N3—Pd1—N6	98.71 (14)

**Table 2:** Hydrogen-bond geometry (Å, °)

*D*—H⋯*A*	*D*—H	H⋯*A*	*D*⋯*A*	*D*—H⋯*A*
C1—H1⋯N3	0.95	2.53	3.044 (5)	114
C1—H1⋯N5^i^	0.95	2.54	3.196 (5)	127
C5—H5⋯N8^ii^	0.95	2.55	3.324 (6)	139
C8—H8⋯N8^iii^	0.95	2.55	3.218 (6)	127
C10—H10⋯N6	0.95	2.51	3.031 (5)	114

Symmetry codes: (i) 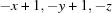; (ii) 
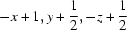
; (iii) 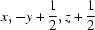.

**Fig. 1.**
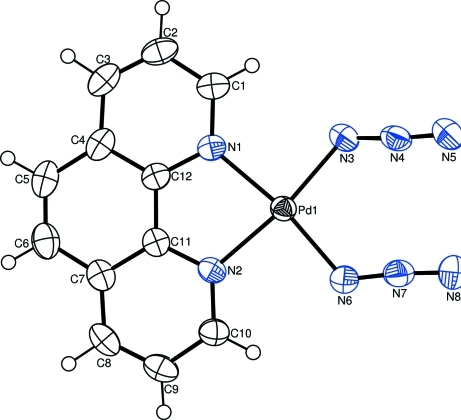
A view of the molecular structure of the title complex, with the atom numbering scheme and displacement ellipsoids drawn at the 50% probability level.

**Fig. 2.**
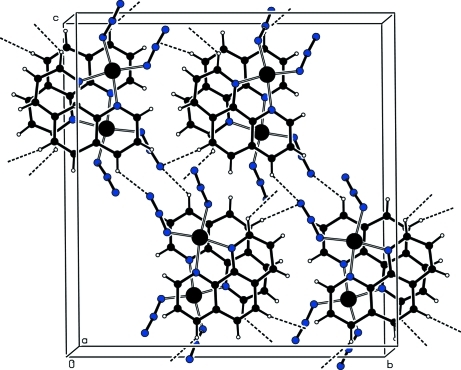
A view of the unit-cell contents of the title complex. Intermolecular C—H···N hydrogen-bond interactions are drawn with dashed lines.

## supplementary crystallographic information

### Comment

Syntheses (Cheng *et al.*, 1977) and crystal structures of Pd^II^ complexes with 1,10-phenanthroline (phen; C_12_H_8_N_2_) and halogenide ions, [Pd*X*_2_(phen)] (*X* = Cl, Br or I), have been reported previously (Ha, 2011*a,b,c*). Here the crystal structure of the pseudohalogenide [Pd(N_3_)_2_(phen)] is reported.

In the title complex, the Pd^II^ ion is four-coordinated in a slightly distorted square-planar environment by two N atoms of the chelating 1,10-phenanthroline ligand and two N atoms from two azide anions (Fig. 1). The main contribution to the distortion is the tight N1—Pd1—N2 chelate angle [81.20 (12)°], which results in a non-linear *trans* arrangement [N1—Pd1—N6 = 170.93 (13)° and N2—Pd1—N3 = 171.39 (13)°]. The Pd—N(phen) bond lengths are slightly longer than the Pd—N(azide) bond lengths [Pd1—N1/2: 2.038 (3) and 2.040 (3) Å; Pd1—N3/6: 2.012 (3) and 2.013 (3) Å] (Table 1). The azido ligands are slightly bent with the bond angles of N3—N4—N5 = 174.8 (4)° and N6—N7—N8 = 174.5 (4)°, however with nearly equal N—N bond lengths [N—N: 1.155 (5)–1.195 (5) Å].

In the crystal, the complex molecules are stacked in columns along the *a* axis and are connected by intermolecular C—H···N hydrogen bonds, forming a three-dimensional network (Fig. 2 and Table 2). In the columns, numerous intermolecular π—π interactions between the six-membered rings are present, the shortest ring centroid-centroid distance being 3.607 (2) Å. Intramolecular C—H···N hydrogen bonds are also present (Table 2).

### Experimental

To a solution of Na_2_PdCl_4_ (0.1475 g, 0.501 mmol) and NaN_3_ (0.3239 g, 4.982 mmol) in MeOH (30 ml) was added 1,10-phenanthroline (0.1044 g, 0.579 mmol), and stirred for 3 h at room temperature. The formed precipitate was separated by filtration, washed with water and acetone, and dried at 323 K, to give a bright yellow powder (0.1615 g). Crystals suitable for X-ray analysis were obtained by slow evaporation from a dimethyl sulfoxide (DMSO) solution at 363 K.

### Refinement

H atoms were positioned geometrically and allowed to ride on their respective parent atoms [C—H = 0.95 Å and *U*_iso_(H) = 1.2*U*_eq_(C)]. The highest peak (1.20 e Å^-3^) and the deepest hole (-0.62 e Å^-3^) in the difference Fourier map are located 1.55 Å and 0.91 Å from the atoms C11 and Pd1, respectively.

### Crystal data

**Table:**

[Pd(N_3_)_2_(C_12_H_8_N_2_)]	*F*(000) = 1456
*M**_r_* = 370.66	*D*_x_ = 1.988 Mg m^−^^3^
Orthorhombic, *P**b**c**a*	Mo *K*α radiation, λ = 0.71073 Å
Hall symbol: -P 2ac 2ab	Cell parameters from 6741 reflections
*a* = 7.0724 (3) Å	θ = 2.5–28.3°
*b* = 18.3069 (7) Å	µ = 1.51 mm^−^^1^
*c* = 19.1309 (7) Å	*T* = 200 K
*V* = 2476.95 (17) Å^3^	Block, yellow
*Z* = 8	0.25 × 0.13 × 0.12 mm

### Data collection

**Table:**

Bruker SMART 1000 CCD diffractometer	3058 independent reflections
Radiation source: fine-focus sealed tube	2244 reflections with *I* > 2σ(*I*)
graphite	*R*_int_ = 0.040
φ and ω scans	θ_max_ = 28.3°, θ_min_ = 2.1°
Absorption correction: multi-scan (*SADABS*; Bruker, 2000)	*h* = −8→9
*T*_min_ = 0.886, *T*_max_ = 1.000	*k* = −24→24
17018 measured reflections	*l* = −25→23

### Refinement

**Table:**

Refinement on *F*^2^	Primary atom site location: structure-invariant direct methods
Least-squares matrix: full	Secondary atom site location: difference Fourier map
*R*[*F*^2^ > 2σ(*F*^2^)] = 0.033	Hydrogen site location: inferred from neighbouring sites
*wR*(*F*^2^) = 0.084	H-atom parameters constrained
*S* = 1.09	*w* = 1/[σ^2^(*F*_o_^2^) + (0.0259*P*)^2^ + 3.9659*P*] where *P* = (*F*_o_^2^ + 2*F*_c_^2^)/3
3058 reflections	(Δ/σ)_max_ = 0.001
190 parameters	Δρ_max_ = 1.20 e Å^−^^3^
0 restraints	Δρ_min_ = −0.62 e Å^−^^3^

### Special details

**Table:**

Geometry. All e.s.d.'s (except the e.s.d. in the dihedral angle between two l.s. planes) are estimated using the full covariance matrix. The cell e.s.d.'s are taken into account individually in the estimation of e.s.d.'s in distances, angles and torsion angles; correlations between e.s.d.'s in cell parameters are only used when they are defined by crystal symmetry. An approximate (isotropic) treatment of cell e.s.d.'s is used for estimating e.s.d.'s involving l.s. planes.
Refinement. Refinement of *F*^2^ against ALL reflections. The weighted *R*-factor *wR* and goodness of fit *S* are based on *F*^2^, conventional *R*-factors *R* are based on *F*, with *F* set to zero for negative *F*^2^. The threshold expression of *F*^2^ > σ(*F*^2^) is used only for calculating *R*-factors(gt) *etc*. and is not relevant to the choice of reflections for refinement. *R*-factors based on *F*^2^ are statistically about twice as large as those based on *F*, and *R*- factors based on ALL data will be even larger.

### Fractional atomic coordinates and isotropic or equivalent isotropic displacement parameters (Å^2^)

**Table:**

	*x*	*y*	*z*	*U*_iso_*/*U*_eq_	
Pd1	0.42841 (4)	0.384720 (15)	0.171291 (14)	0.03115 (10)	
N1	0.3530 (4)	0.48761 (16)	0.20092 (16)	0.0328 (7)	
N2	0.4398 (4)	0.37116 (16)	0.27707 (15)	0.0322 (7)	
N3	0.4003 (5)	0.41288 (19)	0.07018 (17)	0.0443 (8)	
N4	0.4887 (5)	0.38609 (18)	0.02351 (18)	0.0408 (8)	
N5	0.5675 (6)	0.3639 (2)	−0.02492 (19)	0.0547 (10)	
N6	0.5003 (6)	0.27920 (19)	0.15822 (18)	0.0463 (9)	
N7	0.5375 (5)	0.25334 (19)	0.10364 (19)	0.0479 (9)	
N8	0.5816 (8)	0.2236 (2)	0.0529 (2)	0.0781 (15)	
C1	0.3151 (5)	0.5454 (2)	0.1609 (2)	0.0391 (9)	
H1	0.3208	0.5405	0.1115	0.047*	
C2	0.2672 (6)	0.6127 (2)	0.1898 (2)	0.0450 (10)	
H2	0.2401	0.6529	0.1600	0.054*	
C3	0.2592 (6)	0.6212 (2)	0.2605 (2)	0.0461 (10)	
H3	0.2269	0.6673	0.2800	0.055*	
C4	0.2989 (5)	0.5615 (2)	0.3045 (2)	0.0397 (9)	
C5	0.2938 (6)	0.5635 (2)	0.3793 (2)	0.0465 (10)	
H5	0.2607	0.6076	0.4023	0.056*	
C6	0.3351 (6)	0.5037 (2)	0.4177 (2)	0.0472 (10)	
H6	0.3288	0.5068	0.4673	0.057*	
C7	0.3880 (5)	0.4360 (2)	0.3861 (2)	0.0391 (9)	
C8	0.4377 (6)	0.3725 (3)	0.4225 (2)	0.0452 (10)	
H8	0.4366	0.3722	0.4721	0.054*	
C9	0.4880 (6)	0.3106 (2)	0.3863 (2)	0.0461 (10)	
H9	0.5228	0.2675	0.4107	0.055*	
C10	0.4876 (6)	0.3116 (2)	0.3135 (2)	0.0398 (9)	
H10	0.5223	0.2685	0.2889	0.048*	
C11	0.3921 (5)	0.4327 (2)	0.31269 (19)	0.0330 (8)	
C12	0.3466 (5)	0.49549 (19)	0.27182 (19)	0.0330 (8)	

### Atomic displacement parameters (Å^2^)

**Table:**

	*U*^11^	*U*^22^	*U*^33^	*U*^12^	*U*^13^	*U*^23^
Pd1	0.03266 (17)	0.03068 (16)	0.03012 (16)	−0.00224 (12)	0.00067 (12)	0.00230 (11)
N1	0.0282 (16)	0.0314 (16)	0.0388 (16)	−0.0041 (13)	0.0002 (13)	0.0026 (13)
N2	0.0284 (16)	0.0370 (17)	0.0313 (15)	−0.0040 (13)	0.0022 (12)	0.0056 (13)
N3	0.053 (2)	0.047 (2)	0.0330 (18)	0.0048 (17)	−0.0011 (16)	0.0055 (15)
N4	0.049 (2)	0.0395 (18)	0.0344 (18)	−0.0017 (16)	−0.0096 (16)	0.0081 (15)
N5	0.070 (3)	0.059 (2)	0.035 (2)	0.011 (2)	0.0021 (18)	0.0054 (17)
N6	0.064 (2)	0.0362 (18)	0.038 (2)	0.0020 (17)	0.0047 (17)	0.0028 (15)
N7	0.065 (3)	0.0349 (18)	0.044 (2)	0.0002 (17)	−0.0084 (18)	0.0033 (16)
N8	0.137 (5)	0.052 (3)	0.046 (2)	0.019 (3)	−0.008 (3)	−0.010 (2)
C1	0.031 (2)	0.037 (2)	0.049 (2)	−0.0024 (16)	−0.0033 (17)	0.0074 (18)
C2	0.032 (2)	0.035 (2)	0.068 (3)	0.0002 (17)	−0.0020 (19)	0.0091 (19)
C3	0.036 (2)	0.033 (2)	0.070 (3)	−0.0016 (18)	0.004 (2)	−0.0076 (19)
C4	0.029 (2)	0.035 (2)	0.055 (2)	−0.0043 (16)	0.0066 (17)	−0.0083 (18)
C5	0.040 (2)	0.046 (2)	0.053 (3)	−0.0063 (19)	0.009 (2)	−0.017 (2)
C6	0.042 (2)	0.059 (3)	0.041 (2)	−0.010 (2)	0.0051 (18)	−0.009 (2)
C7	0.033 (2)	0.048 (2)	0.037 (2)	−0.0090 (17)	0.0038 (16)	0.0000 (17)
C8	0.041 (2)	0.065 (3)	0.030 (2)	−0.007 (2)	0.0000 (17)	0.0056 (19)
C9	0.044 (2)	0.052 (3)	0.041 (2)	−0.006 (2)	−0.0018 (19)	0.016 (2)
C10	0.040 (2)	0.038 (2)	0.041 (2)	−0.0007 (18)	0.0014 (17)	0.0057 (17)
C11	0.0246 (19)	0.039 (2)	0.0354 (19)	−0.0052 (15)	0.0025 (14)	−0.0003 (16)
C12	0.0259 (18)	0.0317 (18)	0.041 (2)	−0.0063 (15)	0.0002 (15)	0.0015 (16)

### Geometric parameters (Å, °)

**Table:**

Pd1—N1	2.038 (3)	C3—C4	1.407 (6)
Pd1—N2	2.040 (3)	C3—H3	0.9500
Pd1—N3	2.012 (3)	C4—C12	1.402 (5)
Pd1—N6	2.013 (3)	C4—C5	1.432 (6)
N1—C1	1.333 (5)	C5—C6	1.350 (6)
N1—C12	1.365 (5)	C5—H5	0.9500
N2—C10	1.338 (5)	C6—C7	1.430 (6)
N2—C11	1.360 (5)	C6—H6	0.9500
N3—N4	1.195 (5)	C7—C8	1.399 (6)
N4—N5	1.155 (5)	C7—C11	1.407 (5)
N6—N7	1.176 (5)	C8—C9	1.374 (6)
N7—N8	1.156 (5)	C8—H8	0.9500
C1—C2	1.392 (5)	C9—C10	1.392 (5)
C1—H1	0.9500	C9—H9	0.9500
C2—C3	1.363 (6)	C10—H10	0.9500
C2—H2	0.9500	C11—C12	1.427 (5)
			
N1—Pd1—N2	81.20 (12)	C12—C4—C5	118.2 (4)
N3—Pd1—N6	98.71 (14)	C3—C4—C5	125.0 (4)
N3—Pd1—N1	90.27 (13)	C6—C5—C4	121.3 (4)
N6—Pd1—N1	170.93 (13)	C6—C5—H5	119.4
N3—Pd1—N2	171.39 (13)	C4—C5—H5	119.4
N6—Pd1—N2	89.80 (13)	C5—C6—C7	121.9 (4)
C1—N1—C12	118.7 (3)	C5—C6—H6	119.0
C1—N1—Pd1	128.8 (3)	C7—C6—H6	119.0
C12—N1—Pd1	112.5 (2)	C8—C7—C11	117.1 (4)
C10—N2—C11	118.5 (3)	C8—C7—C6	125.2 (4)
C10—N2—Pd1	128.8 (3)	C11—C7—C6	117.7 (4)
C11—N2—Pd1	112.7 (2)	C9—C8—C7	120.0 (4)
N4—N3—Pd1	124.1 (3)	C9—C8—H8	120.0
N5—N4—N3	174.8 (4)	C7—C8—H8	120.0
N7—N6—Pd1	123.6 (3)	C8—C9—C10	119.5 (4)
N8—N7—N6	174.5 (5)	C8—C9—H9	120.2
N1—C1—C2	121.6 (4)	C10—C9—H9	120.2
N1—C1—H1	119.2	N2—C10—C9	122.1 (4)
C2—C1—H1	119.2	N2—C10—H10	118.9
C3—C2—C1	120.3 (4)	C9—C10—H10	118.9
C3—C2—H2	119.9	N2—C11—C7	122.7 (3)
C1—C2—H2	119.9	N2—C11—C12	116.7 (3)
C2—C3—C4	119.8 (4)	C7—C11—C12	120.6 (4)
C2—C3—H3	120.1	N1—C12—C4	122.8 (3)
C4—C3—H3	120.1	N1—C12—C11	116.9 (3)
C12—C4—C3	116.8 (4)	C4—C12—C11	120.3 (3)
			
N3—Pd1—N1—C1	−3.1 (3)	C7—C8—C9—C10	−0.5 (6)
N2—Pd1—N1—C1	178.1 (3)	C11—N2—C10—C9	0.6 (6)
N3—Pd1—N1—C12	178.3 (3)	Pd1—N2—C10—C9	−179.8 (3)
N2—Pd1—N1—C12	−0.5 (2)	C8—C9—C10—N2	0.1 (6)
N6—Pd1—N2—C10	2.4 (3)	C10—N2—C11—C7	−1.0 (5)
N1—Pd1—N2—C10	−178.7 (3)	Pd1—N2—C11—C7	179.3 (3)
N6—Pd1—N2—C11	−178.0 (3)	C10—N2—C11—C12	178.5 (3)
N1—Pd1—N2—C11	0.9 (2)	Pd1—N2—C11—C12	−1.2 (4)
N6—Pd1—N3—N4	−30.7 (4)	C8—C7—C11—N2	0.6 (5)
N3—Pd1—N6—N7	9.6 (4)	C6—C7—C11—N2	−179.9 (3)
N2—Pd1—N6—N7	−171.7 (4)	C8—C7—C11—C12	−178.9 (3)
C12—N1—C1—C2	−0.8 (5)	C6—C7—C11—C12	0.6 (5)
Pd1—N1—C1—C2	−179.3 (3)	C1—N1—C12—C4	1.0 (5)
N1—C1—C2—C3	0.4 (6)	Pd1—N1—C12—C4	179.7 (3)
C1—C2—C3—C4	−0.2 (6)	C1—N1—C12—C11	−178.7 (3)
C2—C3—C4—C12	0.4 (6)	Pd1—N1—C12—C11	0.0 (4)
C2—C3—C4—C5	−179.5 (4)	C3—C4—C12—N1	−0.8 (6)
C12—C4—C5—C6	0.5 (6)	C5—C4—C12—N1	179.1 (3)
C3—C4—C5—C6	−179.6 (4)	C3—C4—C12—C11	178.9 (3)
C4—C5—C6—C7	0.8 (6)	C5—C4—C12—C11	−1.2 (5)
C5—C6—C7—C8	178.1 (4)	N2—C11—C12—N1	0.8 (5)
C5—C6—C7—C11	−1.3 (6)	C7—C11—C12—N1	−179.7 (3)
C11—C7—C8—C9	0.1 (6)	N2—C11—C12—C4	−178.9 (3)
C6—C7—C8—C9	−179.3 (4)	C7—C11—C12—C4	0.6 (5)

### Hydrogen-bond geometry (Å, °)

**Table:**

*D*—H···*A*	*D*—H	H···*A*	*D*···*A*	*D*—H···*A*
C1—H1···N3	0.95	2.53	3.044 (5)	114
C1—H1···N5^i^	0.95	2.54	3.196 (5)	127
C5—H5···N8^ii^	0.95	2.55	3.324 (6)	139
C8—H8···N8^iii^	0.95	2.55	3.218 (6)	127
C10—H10···N6	0.95	2.51	3.031 (5)	114

Symmetry codes: (i) −*x*+1, −*y*+1, −*z*; (ii) −*x*+1, *y*+1/2, −*z*+1/2; (iii) *x*, −*y*+1/2, *z*+1/2.
